# Publisher Correction: Cytoplasmic localization of GRHL3 upon epidermal differentiation triggers cell shape change for epithelial morphogenesis

**DOI:** 10.1038/s41467-018-07486-2

**Published:** 2018-11-20

**Authors:** Chiharu Kimura-Yoshida, Kyoko Mochida, Masa-aki Nakaya, Takeomi Mizutani, Isao Matsuo

**Affiliations:** 1 Department of Molecular Embryology, Research Institute, Osaka Women’s and Children’s Hospital, Osaka Prefectural Hospital Organization 840, Murodo-cho, Izumi, 594-1101 Osaka, Japan; 2 Department of Molecular Biology, Yokohama City University Graduate School of Medicine, 3-9 Fukuura, Kanazawa-ku, Yokohama, 236-0004 Kanagawa Japan; 3 Department of Life Science and Technology, Faculty of Engineering, Hokkai-Gakuen University, Nishi 11-chome, Minami 26-jo, Chuo-ku, Sapporo, 064-0926 Hokkaido, Japan

Correction to: *Nature Communications* 10.1038/s41467-018-06171-8; published online 3 October 2018

The original version of this Article contained an error in the labelling of Fig. 4. In panel i, the sixth column was incorrectly labelled as NSC23766 negative, and should have been NSC23766 positive. This has now been corrected in both the PDF and HTML versions of the Article.

