# Supplementary material for: Cytoplasmic localization of GRHL3 upon epidermal differentiation triggers cell shape change for epithelial morphogenesis
Source: Nat Commun. 2018 Oct 3;9:4059. doi: 10.1038/s41467-018-06171-8 (PMC6170465; doi:10.1038/s41467-018-06171-8)
Supplement: Supplementary file 1 — Supplementary Information [file 41467_2018_6171_MOESM1_ESM.pdf]

Supplementary Information

Supplementary Figures 1-10, Supplementary Table and Supplementary References

Title

Cytoplasmic localization of GRHL3 upon epidermal differentiation triggers cell shape change for epithelial morphogenesis

by Chiharu Kimura-Yoshida *et al.*

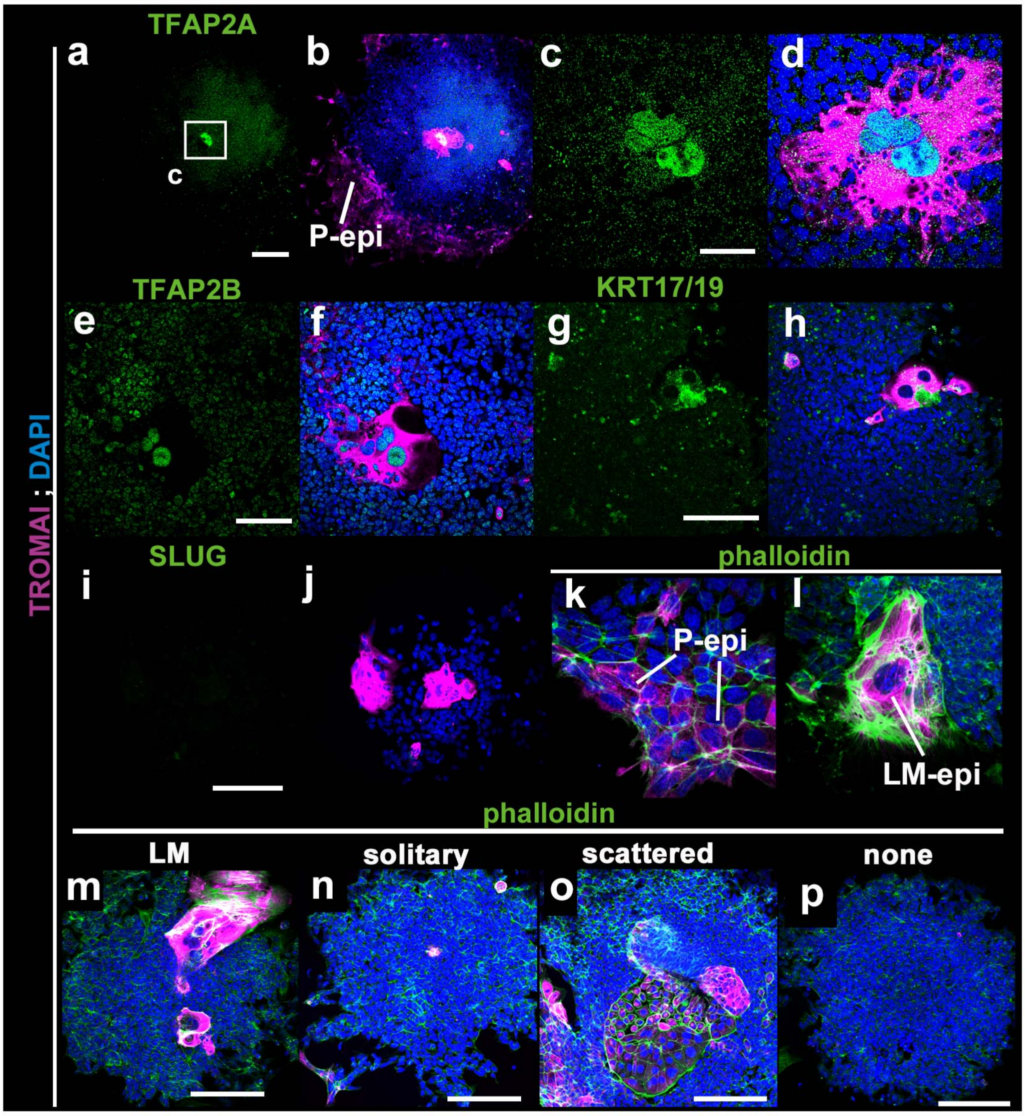

Supplementary Figure 1. Molecular characterization of *Grhl3*-induced large and mature epidermal cells.

(a–l) Immunohistochemical analyses of large and mature (LM)-epidermal cells. TFAP2A (a–d), TFAP2B (e,f), Keratin (KRT) 17/19 (g,h), SLUG (I,j) and F-actin (phalloidin; k,l) are shown in green. Co-immunohistochemistry with anti–TROMA-1 (Keratin-8, magenta) and DAPI (blue; nuclear stain) (a–l). TFAP2A and TFAP2B, two epidermis markers, and keratin-17/19, a mature epidermis marker, were expressed but SLUG, a neural crest marker, was not expressed in *Grhl3*-induced LM-epidermal cells (a–j). TROMA-1 and F-actin are more enriched in LM-epidermal cells (l) than GRHL3-negative epidermal cells formed at the periphery or outside of EBs (b,k, P-epi). (m-p) Examples of TROMA-1 (magenta) positive LM- (m), solitary (n) and scattered (o) epidermal cells are shown. (n) "Solitary" epidermal cells are induced as single cells in the central region of embryoid bodies (EBs) but were not apparently multinucleated. The size of solitary cells is smaller than that of LM-epidermal cells. (o) "Scattered" epidermal cells are induced in numerous numbers in the central region of EBs as aggregates but were not apparently multinucleated. The size of scattered cells is smaller than that of LM-epidermal cells. (p) "None" indicates no TROMA-1–positive epidermal cells were found in the central region of EBs, with the exception of the periphery or outside of EBs. Scale bars represent 200 μm.

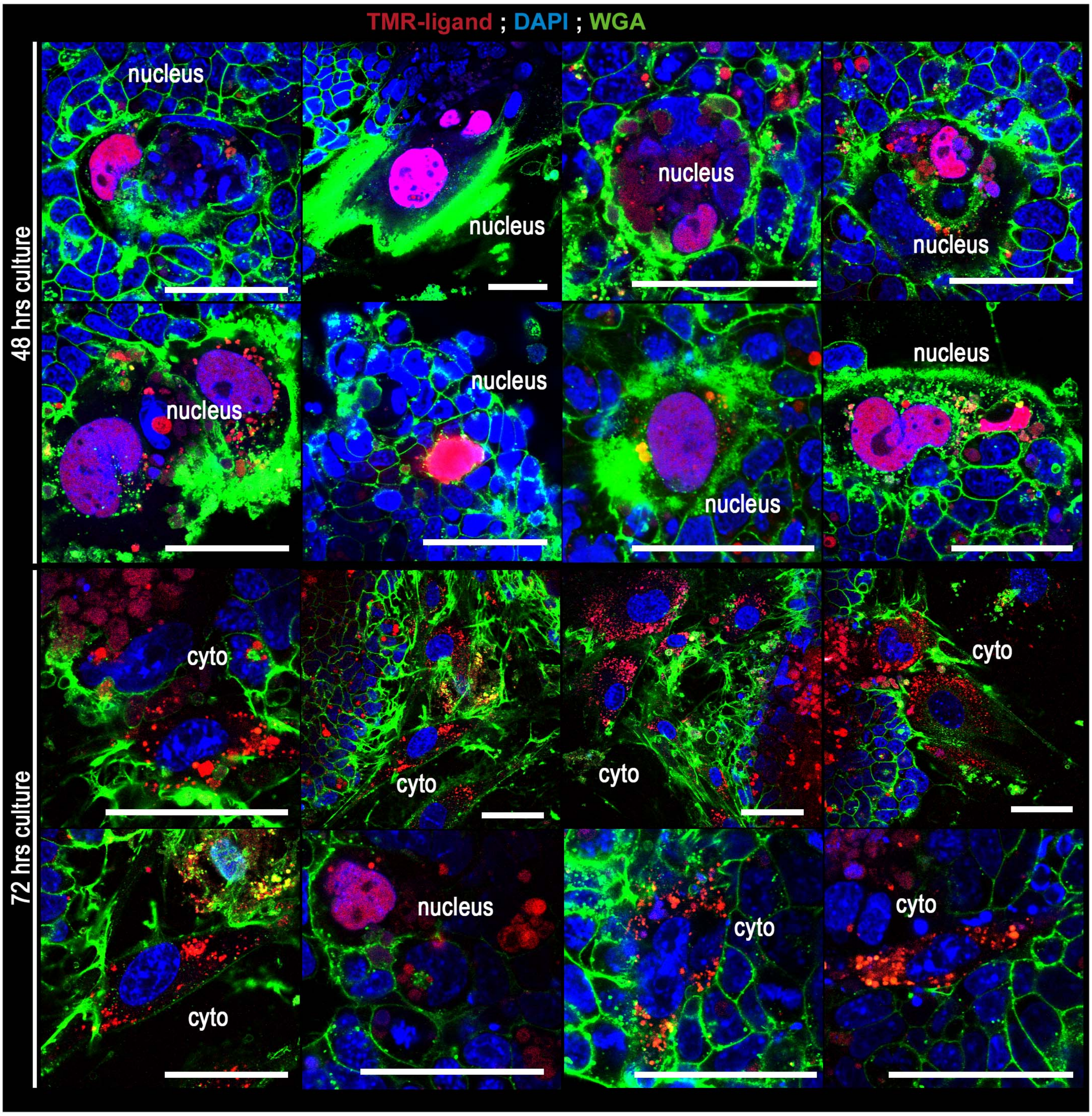

Supplementary Figure 2. Labeling of HaloTagged GRHL3 proteins in living LM-epidermal cells.

Several images of living LM-epidermal cells expressing GRHL3 protein labeled using TMR ligands for quantitative analyses are shown in Fig. 3c. TMR-ligands (GRHL3; red), wheat germ agglutinin (WGA; green) and Hoechst (nuclei; blue), 48 h culture (upper panels) and 72 h culture (lower panels). Scale bars represent 50 µm.

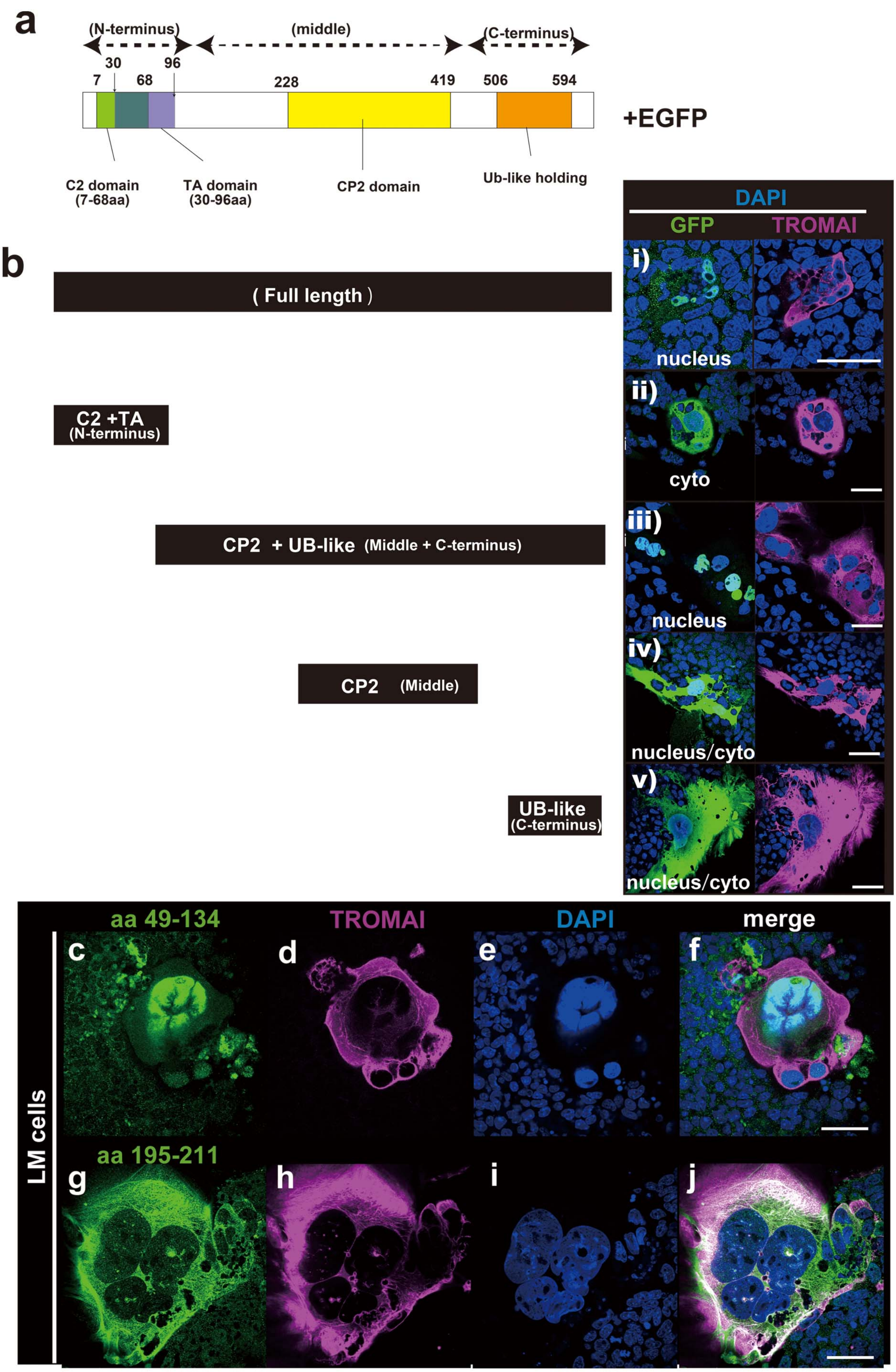

Supplementary Figure 3. Identification of GRHL3 structure contributing to subcellular distribution in LM-epidermal cells.

(a) Schematic illustration showing the protein domain organization of mouse GRHL3. Boxes in green (from 7 aa to 68 aa), purple (from 30 aa to 96 aa), yellow (from 228 aa to 419 aa) and orange (from 506 aa to 594 aa) indicate the C2 domain [1], transcriptional activation (TA) domain, CP2 domain for DNA binding [2], and Ub-like holding domain [3], respectively.
(b) Subcellular localization of five kinds of GRHL3 products: full-length (i), N-terminal (ii), the middle region plus C-terminal (iii), middle region (iv) and C-terminal domains (v) fused to EGFP, respectively, in LM-epidermal cells. TROMA-1 (magenta), EGFP (green) and DAPI (nuclei; blue), respectively. The entire GRHL3 fused to EGFP protein localized to the nucleus exclusively but not to the cytoplasm of LM-epidermal cells (i), which is concordant with endogenous GRHL3 localization as reported previously [4]. The N-terminus (including C2 and TA domains) of GRHL3 fused to EGFP localized to the cytoplasm (ii), whereas the middle region and EGFP-fused C-terminus (including CP2 and Ub-like folding domains) of GRHL3 was distributed in the nucleus (iii). Both of the EGFP-fused middle region (including CP-2 domains) and C-terminus (including the Ub-like folding domain) were distributed in both the nucleus and cytoplasm (iv,v). (c–j) Immunocytochemistry of GRHL3 in LM-epidermal cells using two different antibodies against GRHL3; synthetic peptides corresponding to from 49 aa to 134 aa, and from 195 aa to 211 aa. GRHL3-antibodies (green), TROMA-1 (magenta) and DAPI (blue). GRHL3 protein expression with aa 49–134 and aa195–211 antibodies were predominantly found in the nucleus and cytoplasm of LM-epidermal cells, respectively. Scale bars represent 50 µm.

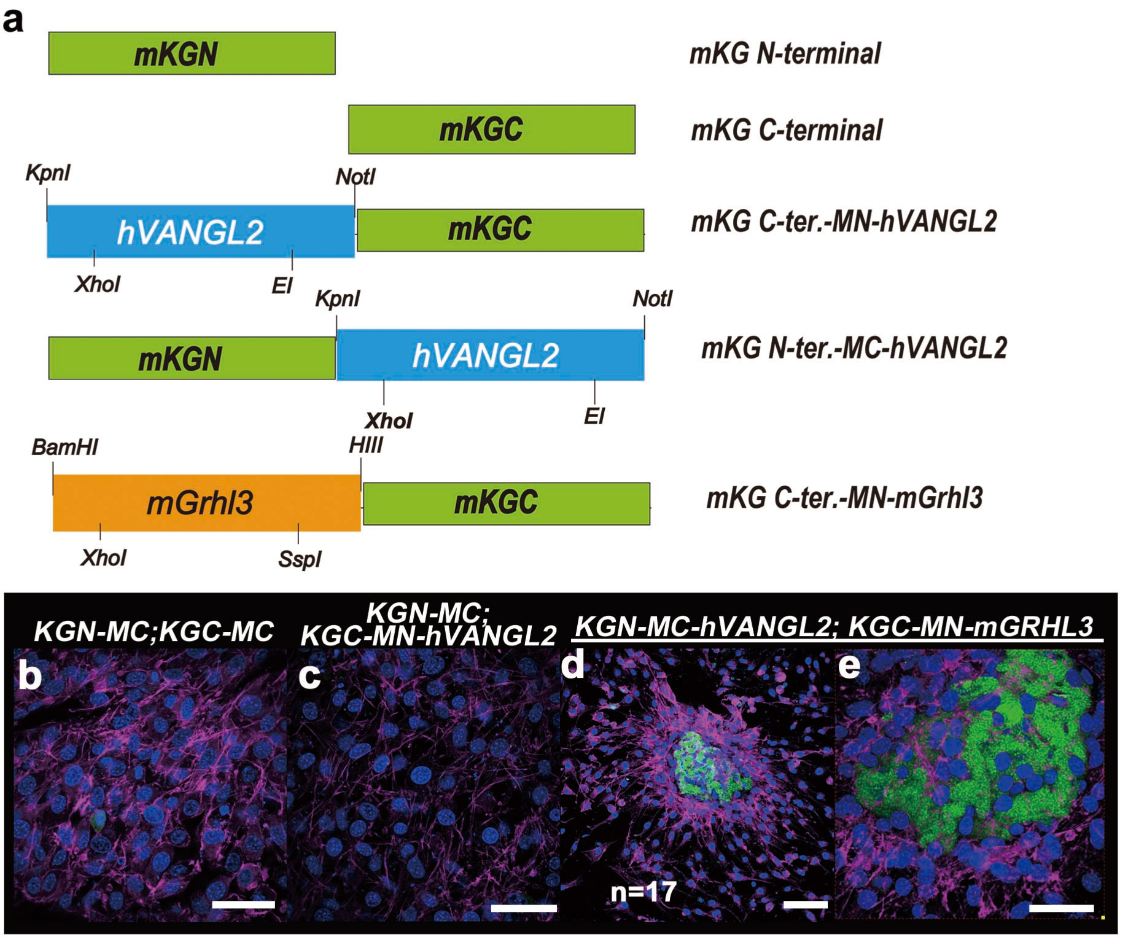

Supplementary Figure 4. Interaction between GRHL3 and VANGL2 as fluorescent signals in mouse fibroblast cells.

(a–e) Protein–protein interaction between mGRHL3 and hVANGL2 in NIH3T3 cells was visualized using a Kusabira–Green reporter system (CoralHueR Fluo-chase Kit; MBL International Corporation, Woburn, MA, USA; cat. no. AM-1100M). Direct visualization was analyzed by confocal fluorescence microscopy. Plasmid structures for the expression of N-terminal or C-terminal mKG fragment–tagged proteins are depicted. Fragment pairs (mKG) without mGRHL3 or hVANGL2 proteins failed to show a fluorescent signal (b,c). Reconstituted mKG fluorescence (green) was solely detected in NIH3T3 cells when the mKG N-terminal fragment fused to hVANGL2, and mGRHL3 fused to the mKG C-terminal fragment were co-expressed (d,e). Scale bars represent 50 (b,c,e) and 100 (d) μm.

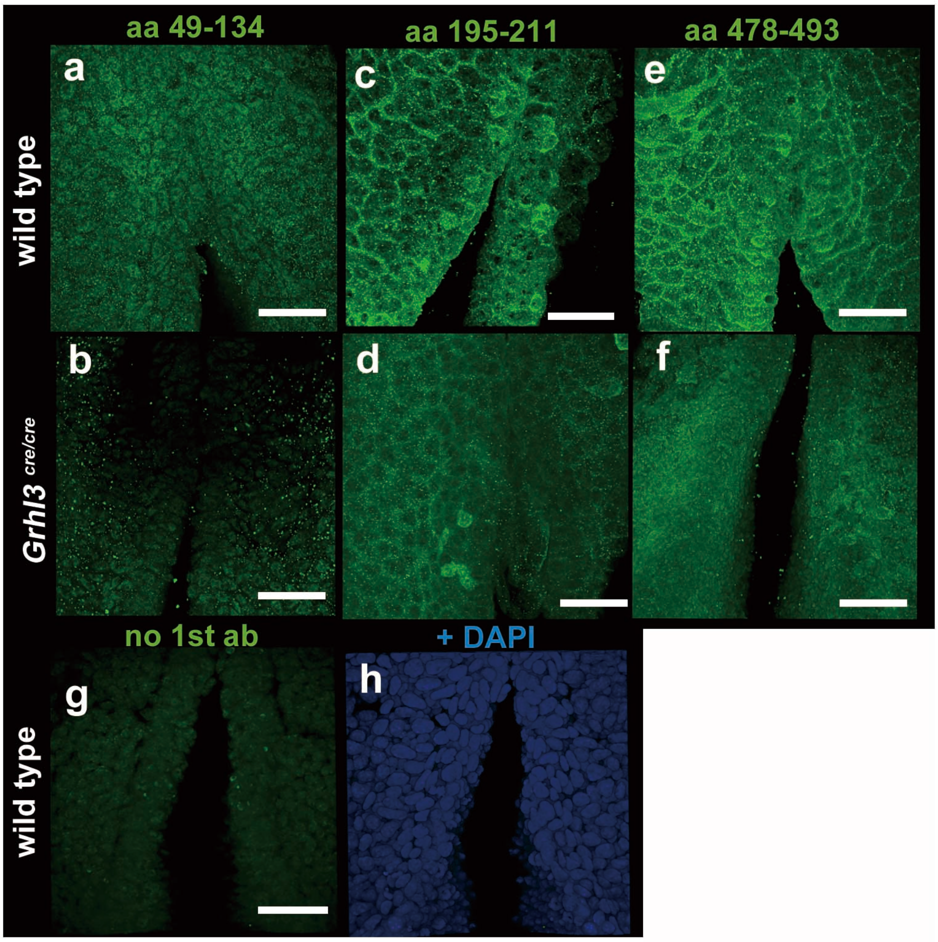

Supplementary Figure 5. Expression analyses with three GRHL3 antibodies during neurulation.

(a–f) Immunohistochemical and fluorescence analyses in wild-type (a,c,e) and *Grhl3*$^{cre/cre}$ (b,d,f) embryos at E8.5. Rabbit polyclonal antibodies against GRHL3 aa 49–134 (a,b), aa 195–211 (c,d) and aa 478–493 (e,f), respectively. GRHL3 (green) and DAPI (dark blue) in a–f. (g,h) The confocal image obtained after not using a primary antibody but using a secondary antibody, Alexa Fluor 488 anti-rabbit (Thermo Fisher Scientific, Waltham, MA, USA; cat. no. A-11034) as a negative control for the wild-type embryo.GRHL3 is expressed mainly at the cell surface and cytoplasm of lateral surface ectoderm and partly in the nucleus of neural folds (c,e). In *Grhl3*$^{cre/cre}$ mutant embryos, some non-specific background staining seems to be present but specific staining, which is found in the wild-type embryos, is convincingly lost with the both antibodies (d,f). Scale bars represent 50 μm.

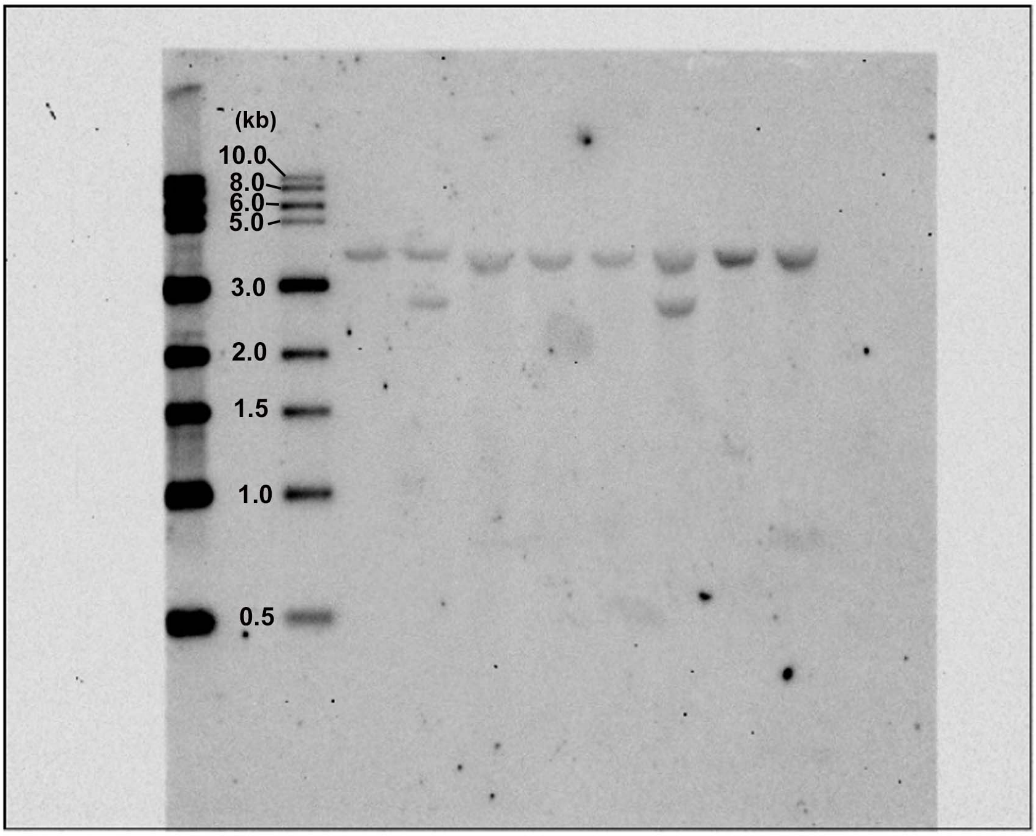

/Users/yoshidachiharu/Desktop/Chemidoc Touch Images geonmic sourthern/2016-04-08 13hr 40min 45sec.scn

Acquisition Information

Imager	ChemiDoc™ Touch
Exposure Time (sec)	580.000 (Signal Accumulation)
Serial Number	732BR0552
Software Version	1.0.0.15
Application	Chemiluminescence
Excitation Source	No Illumination
Emission Filter	No Filter
Binning	4x4

Image Information

Acquisition Date	2016/04/08 13:40
User Name	
Image Area (mm)	X: 166.3 Y: 133.2
Pixel Size (um)	X: 241.3 Y: 241.3
Data Range (Int)	500 - 55890

Supplementary Figure 6. The original chemiluminescence image obtained by southern blot analysis for Figure 7b.

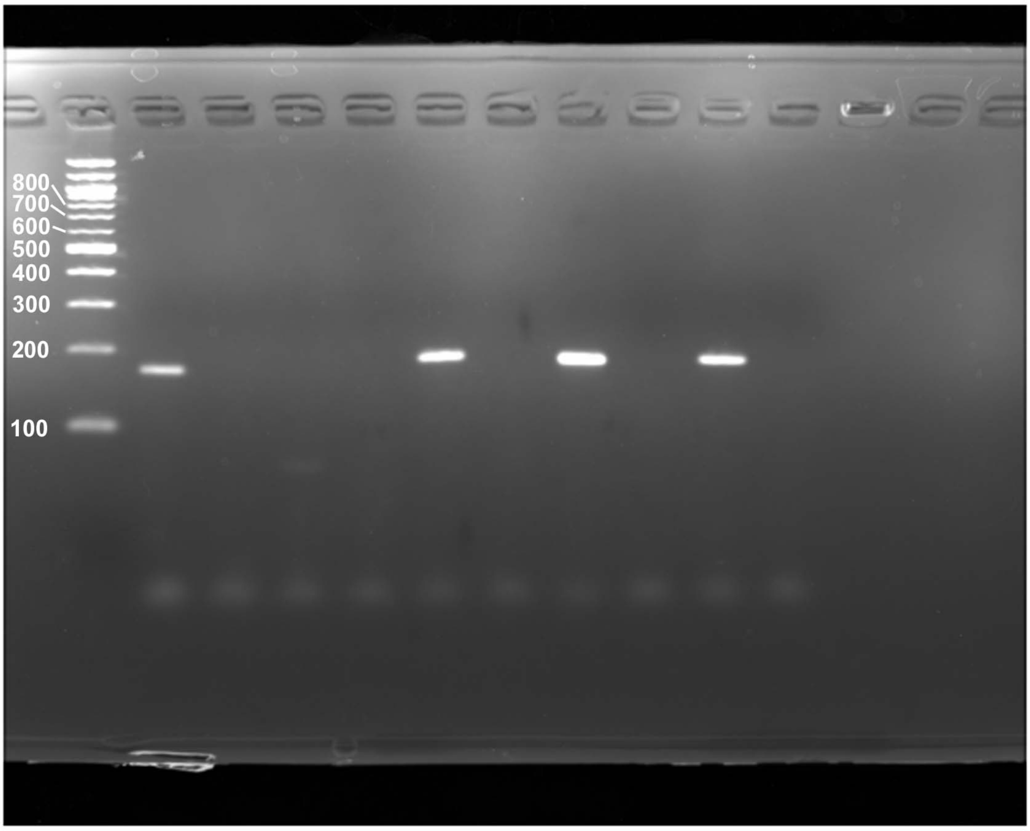

/Users/yoshidachiharu/Desktop/Chemidoc Touch RT-PCR2018-04-04_14.57.30/2018-04-04 13hr 44min 21sec.scn

Acquisition Information

Imager	ChemiDoc™ Touch
Exposure Time (sec)	0.287 (Auto - Intense Bands)
Serial Number	732BR0552
Software Version	1.0.0.15
Application	Ethidium Bromide
Excitation Source	UV Trans illumination
Emission Filter	Standard Filter

Image Information

Acquisition Date	2018/04/04 13:44
User Name	
Image Area (mm)	X: 90.0 Y: 72.1
Pixel Size (um)	X: 32.6 Y: 32.6
Data Range (Int)	177 - 65535

Supplementary Figure 7. The original image obtained by electrophoresis analysis for Figure 7c.

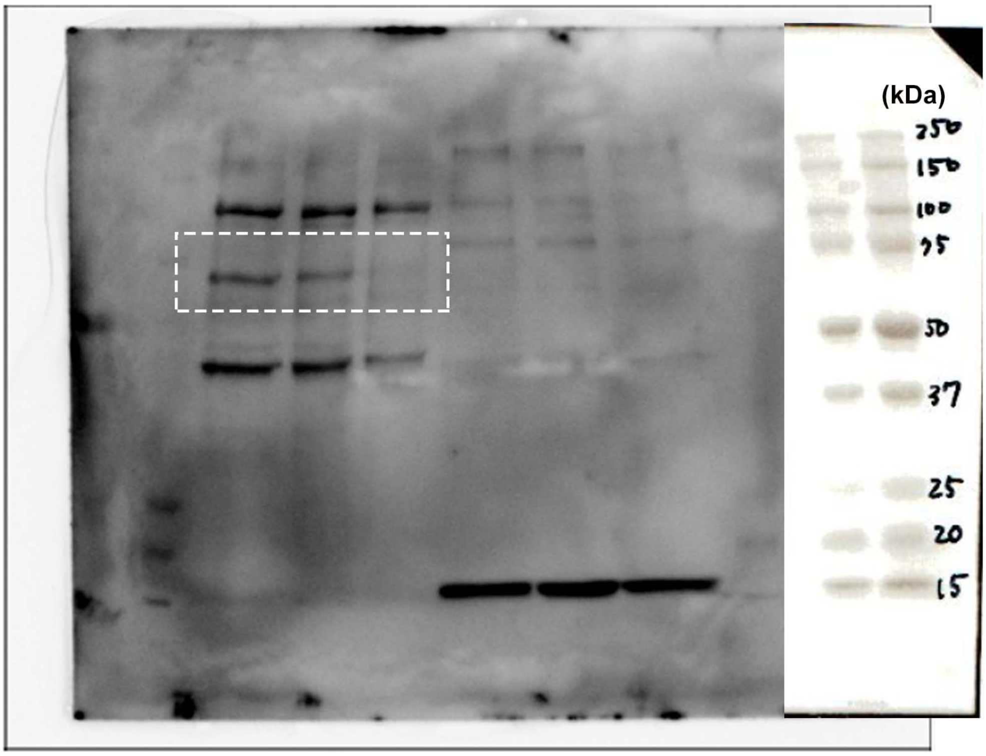

/Volumes/CHIHARU 1GB/Chemidoc Touch Images 2017-07-06_16.39.42/20170623-MBL195-1.5sec.scn

Acquisition Information

Imager	ChemiDoc™ Touch
Exposure Time (sec)	1.500 (Manual)
Serial Number	732BR0552
Software Version	1.0.0.15
Application	Chemiluminescence
Excitation Source	No Illumination
Emission Filter	No Filter
Binning	3x3

Image Information

Acquisition Date	2017/06/23 11:36
User Name	
Image Area (mm)	X: 95.0 Y: 76.1
Pixel Size (um)	X: 103.4 Y: 103.4
Data Range (Int)	601 - 65518

Supplementary Figure 8. The original chemiluminescence image obtained by western blot analysis for Figure 7g.

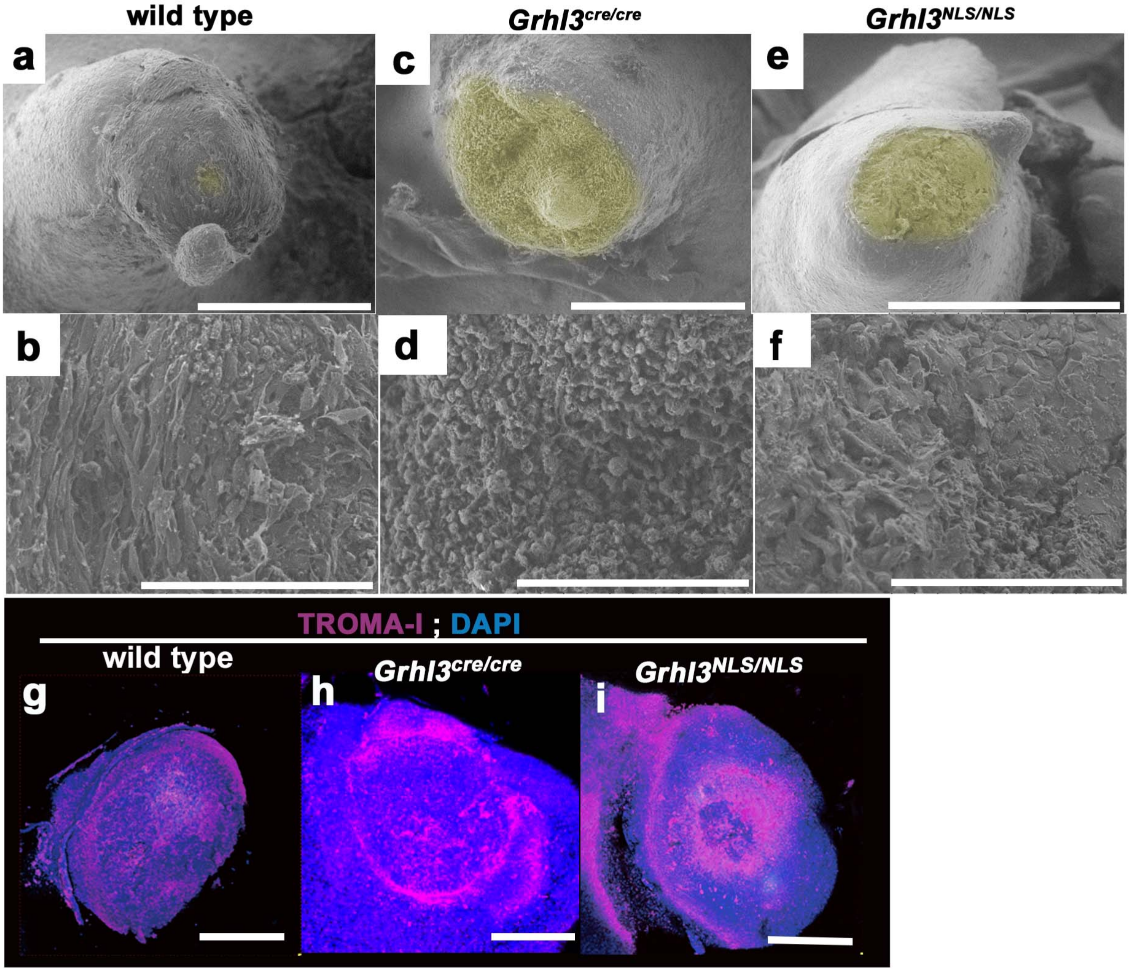

Supplementary Figure 9. Defective wound repair in *Grhl3*$^{NLS/NLS}$ mutant embryos.

(a–e) Scanning electron microscopy (SEM) of wound healing progression of the hind-limb in wild-type (a,b), *Grhl3*$^{cre/cre}$ (c,d) and *Grhl3*$^{NLS/NLS}$ (e,f) mutant embryos at E12.5. The region of disrepair is shown in *Grhl3*$^{cre/cre}$ (c) and *Grhl3*$^{NLS/NLS}$ (e) embryos (yellow). The cells of repaired regions became flattened and elongated toward the edge (b). In contrast, scabrous ectodermal/mesenchymal cells in disrepair were observed in the wound (d,f). (g-i) Whole-mount immunohistochemistry with anti–TROMA-1 (magenta) and DAPI (blue) in hind-limb amputation wounds of wild type (g), *Grhl3*$^{cre/cre}$ (h) and *Grhl3*$^{NLS/NLS}$ embryos (i). Scale bars represent 150 (b,d,f), 400 (g-i), and 500 (a,c,e) µm.

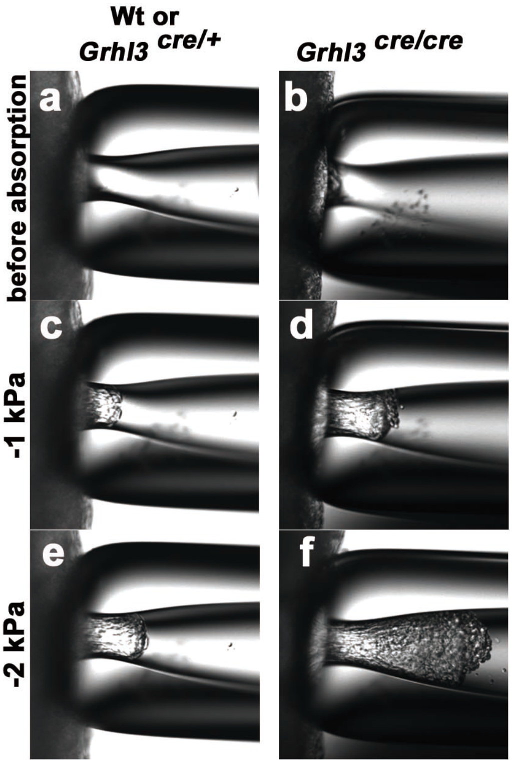

Supplementary Figure 10. Examples of the micropipette–aspiration experiment.

(a–f) Examples of photographic records showing protruded lengths of surface ectoderm (SE) tissues with increasing aspiration pressures of 1 kPa (c,d) and 2 kPa (e,f), respectively. Wild-type or *Grhl3*$^{cre/+}$ (a,c,e) , and *Grhl3*$^{cre/cre}$ (b,d,f) embryos. Average lengths are shown in Fig. 9b (n=10 for wild type or *Grhl3*$^{cre/+}$, n=11 for *Grhl3*$^{cre/cre}$). In a series of 21 experiments, a single identical micropipette was used for aspiration.

Supplementary Table. Antibody and Bioconjugate information

Antibody/Bioconjugate			Markers	Manufacturer	Catalog no.	Source Type	Dilution	Application*
Primary antibodies	TFAP2A (AP2α)		Surface ectoderm/Epidermis	Cell Signaling Technology	#3208	Rabbit IgG	x100	W-I
	TFAP2B (AP2β)		Surface ectoderm/Epidermis	Cell Signaling Technology	#2509	Rabbit IgG	x100	W-I
	β-gal		(Reporter gene)	abcam	ab9361	Chick IgY	x500	W-I,
	CELSR I		PCP-related molecule	Millipore	ABT119	rebbit IgG	x100	W-I
	Claudin4		Epitherial cells tight junctions	abcam	ab53156	Rabbit IgG	x100	W-I
	GFP		(Reporter gene)	abcam	ab13970	Chick IgY	x500	W-I
	GRHL3	aa 49-134	Surface ectoderm/Epidermis (nucleus)	ThermoFisher	PA5-66299	Rabbit IgG	x100	W-I
		aa 195-211	Surface ectoderm/Epidermis (cyto)	MBL(contract)		Rabbit IgG	x100	W-I, WB
		aa 478-493	Surface ectoderm/Epidermis (cyto)	Sigma (contract)		Rabbit IgG	x100	W-I
	HaloTag		Tag	promega	G9281	Rabbit IgG	x100	W-I
	Keratin17/19		Surface ectoderm/epidermis	Cell Signaling Technology	#3984	Rabbit IgG	x100	W-I
	N-cadherin		Neural ectoderm	BD Transduction Laboratory	610920	Mouse IgG1	x200	W-I
	pMLC(Ser19)		myosin light chain 2(smooth muscle) only when phosphorylated at serine 19	Cell Signaling Technology	#3671	Rabbit IgG	x100	W-I
	RhoA (26C4)		Mediator of the PCP pathway	ThermoFisher	sc-418	Mouse IgG1	x100	W-I
	SCRIB		PCP-related molecule	ThermoFisher	PA5-28628	Rabbit IgG	x100	W-I
	SLUG(C19G7)		Neural crest cells	Cell Signaling Technology	#9585	Rabbit IgG	x100	W-I
	TROMA-I (Keratin-8)		Surface ectoderm/Epidermis	Developmental Studies Hybridoma Bank	(TROMA Ic)	Rat IgG	x200	W-I
	VANGL2		PCP-related molecule	Santa Cruz Technology	sc-67136	Rabbit IgG	x100	W-I
Bio-conjugates	phalloidin		F-actin	ThermoFisher	A12379 (Alexa488)		x40	W-I
					A12380 (Alexa568)		x40	W-I
	WGA		Cell membrane	ThermoFisher	W11261		x200	W-I

*Application: W-I; Whole mount-immunohistochemistry/immunofluorescence, WB; Western Blotting.

Supplementary References

1. Zhang, D. & Aravind, L. Identification of novel families and classification of the C2 domain superfamily elucidate the origin and evolution of membrane targeting activities in eukaryotes. *Gene* **469**, 18-30 (2010).
2. Wang, S. & Samakovlis, C. Grainyhead and its target genes in epithelial morphogenesis and wound healing. *Curr. Top. Dev. Biol.* **98**, 35-63 (2012).
3. Kokoszynska, K., Ostrowski, J., Rychlewski, L. & Wyrwicz, L.S. The fold recognition of CP2 transcription factors gives new insights into the function and evolution of tumor suppressor protein p53. *Cell cycle* **7**, 2907-2915 (2008).
4. Boglev, Y. *et al.* The unique and cooperative roles of the Grainy head-like transcription factors in epidermal development reflect unexpected target gene specificity. *Developmental biology* **349**, 512-522 (2011).
